# Supplementary material for: Association between nocturia and serum albumin in the U.S. adults from NHANES 2005–2012
Source: PLoS One. 2024 Aug 15;19(8):e0308980. doi: 10.1371/journal.pone.0308980 (PMC11326611; doi:10.1371/journal.pone.0308980)
Supplement: Attachment — Submitted filename: Manuscript.docx [file pone.0308980.s001.docx]

**Association between nocturia and serum albumin in the U.S. adults** [**from NHANES 2005-2012.**](https://pubmed.ncbi.nlm.nih.gov/37655985/)

Author list: Yangtao Jia^1^, Fangzheng Yang^2^, Xinke Dong^3^, Libin Zhou^2*^ Huimin Long^1*^

Yangtao Jia^1^, The Affiliated Lihuili hospital of Ningbo University, Ningbo, Zhejiang, People's Republic of China

Fangzheng Yang^2^, The Affiliated Lihuili hospital of Ningbo University, Ningbo, Zhejiang, People's Republic of China

Xinke Dong^3^, The Affiliated Lihuili hospital of Ningbo University, Ningbo, Zhejiang, People's Republic of China

Huimin Long^1*^, The Affiliated Lihuili hospital of Ningbo University, Ningbo, Zhejiang, People's Republic of China

Libin Zhou^2*^, The Affiliated Lihuili hospital of Ningbo University, Ningbo, Zhejiang, People’s Republic of China

***Correspondence:**

Huimin Long^1^**^*^**

Email: lhllonghuimin@nbu.edu.cn

Libin Zhou^2^**^*^**

Email: [zlburo2013@sina.com](mailto:zlburo2013@sina.com)

**Keywords:** NHANES, nocturia, serum albumin, cross-section study, U.S. adults.

**Abstract**

**Background:** Nocturia, a prevalent chronic condition, impacts individuals' quality of life but remains underexplored. This study aimed to assess the link between serum albumin levels and nocturia.

**Methods:** Analyzing NHANES data (2005-2012), 6345 adults (≥20 years) were studied. Nocturia was defined as ≥2 nocturnal voiding episodes. Logistic regression and smooth curve fitting analyzed the linear and nonlinear correlations between serum albumin and nocturia, with subgroup analysis.

**Results:** Among 6345 participants, 1821 (28.7%) experienced nocturia. Logistic regression analysis revealed a linear negative correlation between serum albumin and nocturia risk (OR = 0.9549, 95% CI = 0.9280 ~ 0.9827, P = 0.002). Even after quartile division of serum albumin concentration, this correlation persisted within each group, and a smooth curve fitting validated the nonlinear negative correlation between the two. Subgroup analysis further demonstrated significant impacts of BMI, alcohol consumption, and age on this association.

**Conclusion:** This cross-sectional study indicated that higher serum albumin levels were associated with a reduced risk of nocturia in U.S. adults aged 20 and older, highlighting the importance of serum albumin in the prevention and treatment of nocturia and providing clinical guidance.

**Introduction**

Nocturnal voiding, per the definition provided by the International Continence Society (ICS), pertains to the frequency of urination episodes experienced during the main sleep period(1). Manifesting two or more times was considered clinically significant, as this criterion correlates with the quality of life and mortality(2). Increased nocturnal voiding was the most common clinical symptom of lower urinary tract symptom (LUTS), accounting for 33% of all LUTS-related presentations. Its prevalence in young adults aged 20 to 40 years was 11.0%-35.2% in males and 20.4%-43.9% in females(3). Furthermore, this prevalence doubled among older people(4, 5). Additionally, nocturnal voiding was associated with various diseases, including sleep disorders, hypertension, diabetes, and cardiovascular diseases, as well as correlated with overall mortality(3, 6, 7).

Human Serum Albumin (HSA), typically present in concentrations ranging from 3.5 to 5 g/dl, constitutes 50%-60% of the total plasma proteins in healthy adults(8, 9). As a vital carrier and drug-binding protein in plasma, HSA binds and transports both endogenous and exogenous molecules(10). Additionally, albumin maintains balance among intracellular, extracellular and tissue fluids, maintaining osmotic pressure equilibrium, and plays a vital role in inflammation, antioxidant activity, endothelial stability and immune regulation(10-12).

Recent research indicated a higher prevalence of nocturnal voiding in liver cirrhosis patients exhibiting serum albumin levels below the general population average(13). In patients with type 2 diabetes, those with overactive bladder (OAB) and nocturnal voiding had higher serum albumin levels compared to those without(14, 15). These findings implied a certain degree of association between nocturnal voiding and serum albumin levels, although the exact mechanisms and direct relationship remained unclear. Moreover, cross-sectional studies with large samples investigating the current relationship between nocturnal voiding and albumin levels do not exist in populations. For these reasons, we conducted a comprehensive study to explore the relationship within a large population of adults aged 20 and above in the United States.

**Materials and Methods**

**2.1 Population and design**

The National Health and Nutrition Examination Survey (NHANES) was a biennial cross-sectional survey conducted in the United States by the National Center for Health Statistics. This survey systematically gathered representative data on US citizens' health and nutritional status to evaluate the population's overall health and nutritional well-being. Our research utilized integrated demographic, examination, laboratory, and questionnaire data from NHANES from 2005 to 2012. These comprehensive datasets were available for downloading on the NHANES website (https://www.cdc.gov/nchs/nhanes/index.htm). This study specifically explored the correlation between serum albumin levels and nocturia in NHANES participants from 2005 to 2012, involving a cohort of 40790 individuals. Participants were excluded if they were: (1) diagnosed with prostate cancer; (2) had prostate surgery; (3) had a rectal examination in the last seven days; (4) underwent prostate biopsy in the last four weeks; (5) diagnosed with infection or inflammation; (6) underwent cystoscopy in the last four weeks; (7) had missing data on nocturnal voiding or albumin; (8) had incomplete covariate information; (9) were under the age of 20. (10) pregnant and lactating women; (11) had used diuretics such as spironolactone, furosemide, and hydrochlorothiazide for one month. Following rigorous screening, 6345 participants were included in this study.

**2.2 Exposure and outcome**

Participants were screened for nocturia using a structured questionnaire. The inquiry posed was, "During the past 30 days, how many times per night did you most typically get up to urinate from the time going to bed at night to waking up in the morning?" Responses were stratified into categories: 0 times/night, 1 time/night, 2 times/night, 3 times/night, 4 times/night, and 5 or more times/night. In our investigation, individuals who reported urinating ≥2 times/night were identified as having nocturia.

Researchers conducted hypertension assessments using multiple approaches. Firstly, an affirmative response to the query, "Ever been told by a doctor or other health professional that you had hypertension, also called high blood pressure?" classified individuals as hypertensive. Secondly, participants who took prescriptions for hypertension were categorized as hypertensive. Lastly, individuals registering systolic blood pressure ≥130 mmHg or diastolic blood pressure ≥80 mmHg during the physical examination were also deemed hypertensive(16).

Participants were evaluated for diabetes through diverse methods. Firstly, an affirmative response to the question, "Ever been told by a doctor or health professional that you had diabetes or sugar diabetes?" led to classification as having diabetes. Secondly, participants reporting taking prescriptions for diabetes were labelled as having diabetes. Lastly, individuals with a fasting blood glucose level ≥12.6 mmol/L or a glycated hemoglobin level ≥6.5% in laboratory tests were also considered to have diabetes(17).

The Adult Treatment Panel III (ATP 3) of the National Cholesterol Education Program (NCEP) defines hyperlipidemia as having total cholesterol levels ≥200 mg/dL, triglycerides ≥150 mg/dL, HDL levels ≤40 mg/dL in men or ≤50 mg/dL in women, or LDL levels ≥130 mg/dL(18, 19).

Additionally, depression was evaluated utilizing the nine-item Patient Health Questionnaire (PHQ-9) depression scale, where scores range from 0 to 27. A score of ≥10 indicates clinically significant depressive symptoms(20).

**2.3 Covariates selection**

We included potential confounding factors that may influence the association between nocturia and serum albumin levels in a multivariate model based on previous research and clinical experience. These factors encompass gender, age, race, education level, marital status, family poverty income ratio (PIR), body mass index (BMI), alcohol consumption, serum cotinine concentration, diabetes status, hypertension status, depressive disorder status, and hyperlipemia status. Participants' races were divided into five groups: Non-Hispanic White, Non-Hispanic Black, Mexican American, Other Hispanic, and Other races. Age (20-34 years, 35-49 years, 50-64 years, ≥65 years), education levels (Less than high school, High school graduate, More than high school), family PIR (<1, 1-4, >4), BMI (<25 kg/m2, 25 - 30 kg/m2, ≥30 kg/m2), and serum cotinine concentration (<0.015 ng/mL, 0.015-3 ng/mL, >3 ng/mL) were each classified into three categories. Gender (males, females), marital status (Married/Living with partner, Widowed/Divorced/Separated/Never married), diabetes history (Yes, No), hypertension history(Yes, No), alcohol consumption (Yes, No)， hyperlipemia history (Yes, No), and depressive disorder history (Yes, No)were each categorized into two groups.

**2.4 Statistical analysis**

Because of the complex stratified and cluster sampling design of NHANES, we performed a weighted analysis. NHANES combined eight years of total survey data from 2005 to 2012. The combined weight is expressed as WEIGHT_05-12_ = (1/4) × WTMEC2YR_05-06_ + (1/4) × WTMEC2YR_07-08_ + (1/4) × WTMEC2YR_09-10_ + (1/4) × WTMEC2YR_11-12_

Baseline characteristics are reported with categorical variables and continuous variables. The former was presented with case numbers and percentages, and the latter was expressed as mean ± standard deviation. The baseline characteristics of subjects were categorized according to serum albumin quartiles. We used a multivariate logistic regression model to investigate the association between nocturia and diverse serum albumin levels. Association strength was quantified by odds ratios (OR) with corresponding 95% confidence intervals (CI). The multivariate model comprises model 1 (adjusted for none), model 2 (adjusted for age gender and race), and model 3 (adjusted for age, gender, race, marital status, education level, serum cotinine concentration, alcohol consumption, PIR status, BMI status, diabetes, hypertension, hyperlipemia and depressive disorder). Furthermore, we performed interaction and stratified analyses considering the listed covariates. Statistical analyses were conducted using STATA v16.0 and R project. Statistical significance was set at P<0.05.

**Results**

**3.1. Selection of study population**

Among the 40,790 participants in NHANES from 2005 to 2012, 22692 individuals aged 20 and above participated in this study. Subsequently, 4206 samples with incomplete data on nocturia and albumin were excluded, leaving 18486 participants. Further exclusion of 3282 patients meeting specific criteria resulted in 15204 remaining participants. Finally, samples lacking covariate information were removed, resulting in 6345 participants included in the study. (Figure 1)

**Figure 1** Flow chart illustrating the selection of participants in NHANES from 2005 to 2012

**3.2 Baseline Characteristics**

Participants' characteristics were categorized into four groups according to quartiles of serum albumin concentration. Subsequently, the baseline characteristics of the participants were presented. Table 1 illustrated apparent variations in baseline characteristics among the albumin quartiles. The study included 6,345 adults, with a mean (SE) age of 46.355 years. The serum albumin concentration was 42.594 g/L. Of these, 3292 participants were male, and 3053 were female. Individuals with higher albumin concentrations were more likely to be male, aged 20-34 years, married or cohabiting, with a BMI < 25, and without diabetes, hypertension, or nocturia. (Table 1)

**Table 1** Characteristics of participants with different serum albumin levels in NHANES from 2005 to 2010.

| **Characteristics** | **Q1** | **Q2** | **Q3** | **Q4** | **total** | **P-vaule** |
| --- | --- | --- | --- | --- | --- | --- |
| Age(years) |  |  |  |  |  | 0.928 |
|  | 48.533±0.439 | 48.572±0.485 | 47.113±0.459 | 41.833±0.443 | 46.355±0.231 | |
| Serum albumin(g/L) | |  |  |  |  | <0.001 |
|  | 38.545±0.050 | 41.557±0.017 | 43.451±0.016 | 46.341±0.047 | 42.594±0.049 | |
| **Gender(n%)** |  |  |  |  |  | <0.001 |
| Male | 581 (33.18%) | 656 (44.93%) | 919 (59.33%) | 1136 (71.67%) | 3292 (51.88%); | |
| Female | 1170 (66.82%) | 804 (55.07%) | 630 (40.67%) | 449 (28.33%) | 3053 (48.12%) | |
| **Age(n%)** |  |  |  |  |  | <0.001 |
| 20-34 | 336 (19.19%) | 303 (20.75%) | 365 (23.56%) | 608 (38.36%) | 1612 (25.41%); | |
| 35-49 | 513 (29.3%) | 396 (27.12%) | 444 (28.66%) | 408 (25.74%) | 1761 (27.75%); | |
| 50-64 | 504 (28.78%) | 416 (28.49%) | 443 (28.6%) | 373 (23.53%) | 1736 (27.36%); | |
| ≥65 | 398 (22.73%) | 345 (23.63%) | 297 (19.17%) | 196 (12.37%) | 1236 (19.48%) | |
| **Race (n%)** | |  |  |  |  | <0.001 |
| Mexican American | 283 (16.16%) | 264 (18.08%) | 271 (17.5%) | 306 (19.31%) | 1124 (17.71%); | |
| Other Hispanic | 176 (10.05%) | 131 (8.97%) | 157 (10.14%) | 148 (9.34%) | 612 (9.65%); | |
| Non-Hispanic White | 768 (43.86%) | 695 (47.6%) | 774 (49.97%) | 763 (48.14%) | 3000 (47.28%); | |
| Non-Hispanic Black | 444 (25.36%) | 279 (19.11%) | 231 (14.91%) | 196 (12.37%) | 1150 (18.12%); | |
| Other Race | 80 (4.57%) | 91 (6.23%) | 116 (7.49%) | 172 (10.85%) | 459 (7.23%) | |
| **Education(n%)** |  |  |  |  |  | 0.017 |
| Less than high school | 527 (30.1%) | 393 (26.92%) | 385 (24.85%) | 407 (25.68%) | 1712 (26.98%); | |
| High school | 401 (22.9%) | 333 (22.81%) | 367 (23.69%) | 355 (22.4%) | 1456 (22.95%); | |
| More than high school | 401 (22.9%) | 333 (22.81%) | 367 (23.69%) | 355 (22.4%) | 1456 (22.95%); | |
| **Marital status(n%)** | |  |  |  |  | <0.001 |
| Married/Living with partner | 807 (46.09%) | 564 (38.63%) | 561 (36.22%) | 604 (38.11%) | 2536 (39.97%); | |
| Widowed/Divorced  /Separated/Never married | 944 (53.91%) | 896 (61.37%) | 988 (63.78%) | 981 (61.89%) | 3809 (60.03%) | |
| **PIR(n%)** |  |  |  |  |  | <0.001 |
| ≤1 | 451 (25.76%) | 304 (20.82%) | 288 (18.59%) | 343 (21.64%) | 1386 (21.84%); | |
| 1-4 | 913 (52.14%) | 784 (53.7%) | 815 (52.61%) | 828 (52.24%) | 3340 (52.64%); | |
| ≥4 | 387 (22.1%) | 372 (25.48%) | 446 (28.79%) | 414 (26.12%) | 1619 (25.52%) | |
| **BMI(n%)** |  |  |  |  |  | <0.001 |
| <25 | 301 (17.19%) | 371 (25.41%) | 476 (30.73%) | 664 (41.89%) | 1812 (28.56%); | |
| 25-30 | 493 (28.16%) | 515 (35.27%) | 586 (37.83%) | 580 (36.59%) | 2174 (34.26%); | |
| ≥30 | 957 (54.65%) | 574 (39.32%) | 487 (31.44%) | 341 (21.51%) | 2359 (37.18%) | |
| **Cotinine(ng/mL)(n%)** | |  |  |  |  | 0.041 |
| <0.015 | 338 (19.3%) | 323 (22.12%) | 349 (22.53%) | 337 (21.26%) | 1347 (21.23%); | |
| 0.015-3 | 909 (51.91%) | 730 (50%) | 789 (50.94%) | 759 (47.89%) | 3187 (50.23%); | |
| >3 | 504 (28.78%) | 407 (27.88%) | 411 (26.53%) | 489 (30.85%) | 1811 (28.54%) | |
| **Drink at least 12 drinks/year(n%)** | | |  |  |  | <0.001 |
| No | 595 (33.98%) | 402 (27.53%) | 353 (22.79%) | 292 (18.42%) | 1642 (25.88%); | |
| Yes | 1156 (66.02%) | 1058 (72.47%) | 1196 (77.21%) | 1293 (81.58%) | 4703 (74.12%) | |
| **Diabetes history(n%)** | |  |  |  |  | <0.001 |
| No | 1228 (70.13%) | 1108 (75.89%) | 1256 (81.08%) | 1348 (85.05%) | 4940 (77.86%); | |
| Yes | 523 (29.87%) | 352 (24.11%) | 293 (18.92%) | 237 (14.95%) | 1405 (22.14%) | |
| **Hypertension history(n%)** | |  |  |  |  | <0.001 |
| No | 712 (40.66%) | 661 (45.27%) | 707 (45.64%) | 828 (52.24%) | 2908 (45.83%); | |
| Yes | 1039 (59.34%) | 799 (54.73%) | 842 (54.36%) | 757 (47.76%) | 3437 (54.17%) | |
| **Hyperlipemia(n%)** | |  |  |  |  | <0.001 |
| No | 372 (21.25%) | 401 (27.47%) | 404 (26.08%) | 465 (29.34%) | 1642 (25.88%); | |
| Yes | 1379 (78.75%) | 1059 (72.53%) | 1145 (73.92%) | 1120 (70.66%) | 4703 (74.1%) | |
| **Depressive disorder(n%)** | |  |  |  |  | <0.001 |
| No | 1545 (88.24%) | 1324 (90.68%) | 1439 (92.9%) | 1489 (93.94%) | 5797 (91.36%); | |
| Yes | 206 (11.76%) | 136 (9.32%) | 110 (7.1%) | 96 (6.06%) | 548 (8.64%) | |
| **Nocturia(n%)** |  |  |  |  |  | <0.001 |
| Yes | 1078 (61.56%) | 1037 (71.03%) | 1154 (74.5%) | 1255 (79.18%) | 4524 (71.3%); | |
| No | 673 (38.44%) | 423 (28.97%) | 395 (25.5%) | 330 (20.82%) | 1821 (28.7%) | |
| **Times of nocturia(n%)** | |  |  |  |  | <0.001 |
| 0 | 459 (26.21%) | 450 (30.82%) | 529 (34.15%) | 680 (42.9%) | 2118 (33.38%); | |
| 1 | 619 (35.35%) | 587 (40.21%) | 625 (40.35%) | 575 (36.28%) | 2406 (37.92%); | |
| 2 | 376 (21.47%) | 261 (17.88%) | 254 (16.4%) | 213 (13.44%) | 1104 (17.4%); | |
| 3 | 172 (9.82%) | 113 (7.74%) | 93 (6%) | 74 (4.67%) | 452 (7.12%); | |
| 4 | 77 (4.4%) | 30 (2.05%) | 31 (2%) | 24 (1.51%) | 162 (2.55%); | |
| 5 or more | 48 (2.74%) | 19 (1.3%) | 17 (1.1%) | 19 (1.2%) | 103 (1.62%) | |
| Q1: Serum albumin<40g/L, Q2:40g/L≤PIR<42g/L, Q3: 42g/L≤PIR<44g/L, Q4: PIR≥44g/L. | | | | | | |

**3.3 Albumin was associated with a decrease in nocturia**

Table 2 described the logistic regression analysis results between serum albumin concentration and nocturia. Initially, serum albumin was analyzed as a continuous variable, indicating a negative correlation with the risk of nocturia (OR = 0.9549, 95% CI = 0.9280-0.9827, P = 0.002). Subsequently, serum albumin concentration was divided into quartiles. Compared to quartile 1, individuals in the other quartiles had a decreased odds of experiencing nocturia by 33.07% (OR = 0.7693, 95% CI = 0.6220-0.9514, P = 0.016), 28.98% (OR = 0.7102, 95% CI = 0.5716-0.8825, P = 0.002), and 25.04% (OR = 0.7496, 95% CI = 0.5900-0.9523, P = 0.018), respectively. In all models, serum albumin exhibited a negative correlation with nocturia, with a significant decrease in the risk of nocturia as serum albumin levels increased. Additionally, restricted cubic spline regression was used to assess the dose-response relationship between serum albumin and nocturia in the three models. The study results demonstrated a significant non-linear relationship between albumin and nocturia (P < 0.0001, P for non-linear < 0.0001).

**Table 2** Association between nocturia and albumin.

|  | **Model 1** | **P value** | **Model 2** | **P value** | **Model 3** | **P value** |
| --- | --- | --- | --- | --- | --- | --- |
| **Serum albumin** | |  |  |  |  |  |
|  | 0.8949(0.8739-0.9165) | <0.0001 | 0.9294（0.9040-0.9555） | <0.0001 | 0.9549（0.9280-0.9827） | 0.002 |
| **Serum albumin** |  |  |  |  |  |  |
| Q1 | Reference |  | Reference |  | Reference | |
| Q2 | 0.6584(0.5423-0.7992) | <0.0001 | 0.6811（0.5542-0.8371） | 0.0003 | 0.7693（0.6220-0.9514） | 0.016 |
| Q3 | 0.5181(0.4265-0.6295) | <0.0001 | 0.5925（0.4805-0.7305） | <0.0001 | 0.7102（0.5716-0.8825） | 0.002 |
| Q4 | 0.4373(0.3549-0.5384) | <0.0001 | 0.6120（0.4855-0.7715） | <0.0001 | 0.7496（0.5900-0.9523） | 0.018 |

Model 1 adjusts for none.

Model 2 adjusts for age, ethnicity, and gender.

Model 3 adjusts for age, gender, ethnicity, education, BMI, marital status, PIR status, serum cotinine, alcohol status, hypertension history, diabetes history, hyperlipemia history and Depressive disorder history.

**Figure 2** Relationship between nocturia and albumin in Model 3.

**3.4 Subgroup analysis**

Subgroup analyses were conducted to assess the strength and consistency of the association between serum albumin concentration and nocturia (Figure 3). In model 3 accounting for all relevant adjustments, the correlation between nocturia and serum albumin exhibited significant differences in age, drink and BMI interaction tests (P<0.05). Similarly, subgroup analysis of the correlation between serum albumin concentration and nocturia frequency was conducted in model 3(Figure 4), revealing significant differences across different levels of PIR subgroups (P=0.01).

**Figure 3** Associations of nocturia and serum albumin in various subgroups.

**Figure 4** Association of nocturia frequency and serum albumin in different subpopulations.

**Discussion**

In this study, we enrolled 22692 participants aged 20 years and older to investigate the correlation between serum albumin and nocturia. Although previous studies had explored the aetiology of nocturia, there had been limited research on the association between nocturia and serum albumin. Our results revealed a negative correlation between serum albumin levels and nocturia (urination ≥ 2 times/night), whether treated as a continuous or quartile variable. Importantly, this association remained altered by age BMI and drink. This was the inaugural cross-sectional study employing the NHANES database to scrutinize the relationship between nocturia and serum albumin in a large and diverse population.

There was a growing awareness of nocturia as a highly prevalent chronic condition, meriting in-depth investigation. However, existing treatment modalities were constrained, and diagnostic markers were scarce, resulting in suboptimal treatment and management outcomes for nocturia(1, 21). With the progress in nocturia research, several studies had found a significant link between serum albumin and nocturia. Based on these insights, we conducted a study examining the correlation between albumin and nocturia utilizing the NHANES database. Our findings aligned with prior reports, affirming a negative correlation between serum albumin levels and nocturia. In a recent clinical investigation involving patients with chronic liver cirrhosis in Taiwan, participants exhibited an average serum albumin level of 3.85 g/dl, below the norm for the general population, with 62% of these participants displaying noticeable symptoms of nocturia(13). In a preceding cross-sectional investigation on type Ⅱ diabetes, participants experiencing nocturia exhibited markedly lower serum albumin levels than their counterparts without nocturia.(14) Notably, in additional clinical exploration of type Ⅱ diabetes, individuals with overactive bladder (OAB) manifested a substantial reduction in serum albumin levels relative to those without OAB(15). Drawing from these findings, we posited that serum albumin could indicate the overall state of nocturia, presenting potential as a diagnostic marker in clinical settings and offering guidance for treatment.

However, the precise mechanism linking serum albumin and nocturia remained unclear. We investigated potential pathways by reviewing existing literature. It was widely acknowledged that as the most abundant protein in plasma, albumin played a pivotal role in diverse physiological functions, such as pH regulation in plasma and the modulation of fluid transport(21, 22). Prior investigations had partially revealed the mechanisms associating albumin levels with nocturia. Diminished serum albumin levels could reduce osmotic pressure and fluid accumulation in various bodily regions. Thus, the human body initiates compensatory diuresis to uphold the equilibrium of colloid osmotic pressure (23). Also, maintaining a supine position at night allowed the re-entry of accumulated fluid into circulation, causing an elevation in systolic blood pressure. Concurrently, there was a reduction in serum levels of nocturnal antidiuretic hormones, resulting in increased urine output(24). Secondly, serum albumin levels clinically served as a crucial indicator for evaluating liver function(25, 26). Diminished serum albumin levels signified a reduction in hepatic protein synthesis capacity. Historical research indicated a close correlation between cholinesterase and albumin levels produced by the liver (26, 27). The decline in serum albumin levels signified a decrease in cholinesterase content and implied an increase in cholinergic activity. Additionally, activating the micturition reflex delayed the clearance of a substantial amount of acetylcholine secreted by parasympathetic nerve terminals in the bladder, promoting bladder contraction and voiding(28). These mechanisms proposed that serum albumin might impact bladder spasms and voiding frequency, consequently influencing nocturnal urination. Based on these potential mechanisms and our study findings, increasing patients' serum albumin levels may be beneficial in alleviating nocturia symptoms in clinical practice.

Through subgroup analysis, we found significant interactions between BMI, alcohol consumption, and age in the correlation between nocturia and serum albumin. Previous research and clinical expertise also indicated that age was a significant factor in nocturia, as the prevalence of diseases such as diabetes, obesity, and hypertension increased with age, affecting urinary function and consequently increasing the risk of nocturia(29, 30). These findings validated the credibility of our results. Furthermore, individuals who consumed alcohol exhibited a lower risk of nocturia, possibly due to the improvement of insulin sensitivity and reduction in testosterone levels with mild to moderate alcohol consumption, consistent with previous research(31, 32). However, other studies suggested that moderate to heavy alcohol consumption led to more severe LUTS symptoms, contrary to our experimental conclusions(33, 34). Additionally, previous research indicated that obesity was also a significant influencing factor in nocturia. Obesity could lead to increased intra-abdominal pressure, directly causing nocturia(35). Insulin resistance and hyperinsulinemia caused by obesity could stimulate hypothalamic centers, regulating sympathetic tone, increasing catecholamine levels, promoting prostate growth, and indirectly causing nocturia(36, 37). Studies in Taiwan and Japan had shown a positive correlation between obesity and the risk of nocturia(38, 39). Moreover, we further analyzed the frequency of nocturia and found significant interactions between PIR and the correlation between nocturia frequency and serum albumin. PIR was an essential indicator of income level, and lower household income was associated with poorer household health protection, hindering timely prevention, diagnosis, and treatment of diseases.(40) Many studies also suggested a significant correlation between income level and nocturia.(41) However, due to the cross-sectional nature of the NHANES database, the exact mechanisms by which these factors influenced the association between serum albumin and nocturia still needed to be determined.

This study had several significant strengths. Firstly, using a large sample size enhanced the generalizability of our research findings. Secondly, we conducted a preliminary investigation into the correlation between nocturia and serum albumin using the NHANES database, thereby improving physicians' understanding of serum albumin levels in patients with nocturia. Lastly, we systematically stratified various factors and examined their interactions with nocturia. However, this study had several limitations. Firstly, the assessment of participants' nocturia relied on self-reported questionnaires, and diagnosis depended on participants completing voiding diaries, which were subjective and may have needed to be more accurate. Secondly, our sample size was relatively small, and larger-scale studies were needed in the future to obtain more precise results. Additionally, despite the inclusion of many confounding factors and exclusion criteria, other unconsidered factors may still have affected the experimental results. Furthermore, this study was cross-sectional, only suitable for exploring associations, and could not infer causality. Moreover, based on the current research findings, the exact mechanism by which serum albumin affected nocturia remained unclear, and more prospective studies were needed to validate our hypotheses and discuss the mechanism between nocturia and serum albumin.

**Conclusion**

The association between serum albumin levels and the risk of nocturia is identified. A decrease in serum albumin levels correlates with an increased risk of nocturia, suggesting it can be an excellent indicator for predicting the risk. Although we provide some clinical insights, additional randomized controlled studies will be necessary in the future to provide more evidence for precise prevention and treatment.

**Data availability statement**

The original dataset can be publicly accessed on the NHANES database (https://www.cdc.gov/nchs/nhanes/index.htm). All relevant data are within the manuscript and its Supporting Information files.

**Acknowledgements**

We extend our gratitude to all participants in the US National Health and Nutrition Examination Survey (NHANES) and value the unrestricted access offered to the public by the NHANES database.

**Funding Statement**

The authors disclosed receipt of the following financial support for this article: This work was supported by the Natural Science Foundation of Ningbo Municipality (2021J281), the Key Cultivating Discipline of LihHuiLi Hospital (2022-P09) and Ningbo Key Clinical Speciality Construction Project (2023-BZZ)

**References**

1.	Dani H, Esdaille A, Weiss JP. Nocturia: aetiology and treatment in adults. *Nat Rev Urol*. 2016;13:573-83.doi: 10.1038/nrurol.2016.134

2.	Bryan RT, Zeegers MP. Editorial comment on: Nocturia frequency, bother, and quality of life: how often is too often? A population-based study in Finland. *Eur Urol*. 2010;57:496-7.doi: 10.1016/j.eururo.2009.03.081

3.	Chen M, He W, Cai S, Chen Z, Ye H, Jin Z, et al. Association of nocturia with cardiovascular and all-cause mortality: a prospective cohort study with up to 31 years of follow-up. *Front Public Health*. 2023;11:1292362.doi: 10.3389/fpubh.2023.1292362

4.	Bosch JL, Weiss JP. The prevalence and causes of nocturia. *J Urol*. 2013;189:S86-92.doi: 10.1016/j.juro.2012.11.033

5.	Markland AD, Vaughan CP, Johnson TM, 2nd, Goode PS, Redden DT, Burgio KL. Prevalence of nocturia in United States men: results from the National Health and Nutrition Examination Survey. *J Urol*. 2011;185:998-1002.doi: 10.1016/j.juro.2010.10.083

6.	Kupelian V, Fitzgerald MP, Kaplan SA, Norgaard JP, Chiu GR, Rosen RC. Association of nocturia and mortality: results from the Third National Health and Nutrition Examination Survey. *J Urol*. 2011;185:571-7.doi: 10.1016/j.juro.2010.09.108

7.	Fan Y, Wei F, Lang Y, Qi W. Meta-analysis of nocturia and risk of all-cause mortality in adult population. *Int J Cardiol*. 2015;195:120-2.doi: 10.1016/j.ijcard.2015.03.168

8.	Caraceni P, O'Brien A, Gines P. Long-term albumin treatment in patients with cirrhosis and ascites. *J Hepatol*. 2022;76:1306-17.doi: 10.1016/j.jhep.2022.03.005

9.	Wu N, Liu T, Tian M, Liu C, Ma S, Cao H, et al. Albumin, an interesting and functionally diverse protein, varies from 'native' to 'effective' (Review). *Mol Med Rep*. 2024;29.doi: 10.3892/mmr.2023.13147

10.	Donadio C, Tognotti D, Donadio E. Albumin modification and fragmentation in renal disease. *Clin Chim Acta*. 2012;413:391-5.doi: 10.1016/j.cca.2011.11.009

11.	Rabbani G, Ahn SN. Structure, enzymatic activities, glycation and therapeutic potential of human serum albumin: A natural cargo. *Int J Biol Macromol*. 2019;123:979-90.doi: 10.1016/j.ijbiomac.2018.11.053

12.	Duran-Guell M, Flores-Costa R, Casulleras M, Lopez-Vicario C, Titos E, Diaz A, et al. Albumin protects the liver from tumor necrosis factor alpha-induced immunopathology. *FASEB J*. 2021;35:e21365.doi: 10.1096/fj.202001615RRR

13.	Chuang PH, Chang YH, Hsiao PJ, Chou EC. Diagnostic Potential of Low Serum Platelet, Albumin and Prolong PT-INR for Overactive Bladder and Nocturia in Chronic Hepatitis-Related Liver Cirrhosis. *J Clin Med*. 2021;10.doi: 10.3390/jcm10132838

14.	Liu RT, Chung MS, Chuang YC, Lee JJ, Lee WC, Chang HW, et al. The presence of overactive bladder wet increased the risk and severity of erectile dysfunction in men with type 2 diabetes. *J Sex Med*. 2012;9:1913-22.doi: 10.1111/j.1743-6109.2012.02738.x

15.	Afsar B, Elsurer R. Central hemodynamics, vascular stiffness, and nocturia in patients with type 2 diabetes. *Ren Fail*. 2015;37:359-65.doi: 10.3109/0886022X.2015.1088335

16.	Whelton PK, Carey RM, Aronow WS, Casey DE, Jr., Collins KJ, Dennison Himmelfarb C, et al. 2017 ACC/AHA/AAPA/ABC/ACPM/AGS/APhA/ASH/ASPC/NMA/PCNA Guideline for the Prevention, Detection, Evaluation, and Management of High Blood Pressure in Adults: Executive Summary: A Report of the American College of Cardiology/American Heart Association Task Force on Clinical Practice Guidelines. *Circulation*. 2018;138:e426-e83.doi: 10.1161/CIR.0000000000000597

17.	ElSayed NA, Aleppo G, Aroda VR, Bannuru RR, Brown FM, Bruemmer D, et al. 2. Classification and Diagnosis of Diabetes: Standards of Care in Diabetes-2023. *Diabetes Care*. 2023;46:S19-S40.doi: 10.2337/dc23-S002

18.	Mahemuti N, Jing X, Zhang N, Liu C, Li C, Cui Z, et al. Association between Systemic Immunity-Inflammation Index and Hyperlipidemia: A Population-Based Study from the NHANES (2015-2020). *Nutrients*. 2023;15.doi: 10.3390/nu15051177

19.	Yan S, Luo W, Lei L, Zhang Q, Xiu J. Association between serum Klotho concentration and hyperlipidemia in adults: a cross-sectional study from NHANES 2007-2016. *Front Endocrinol (Lausanne)*. 2023;14:1280873.doi: 10.3389/fendo.2023.1280873

20.	Chen J, Liu Z, Yang L, Zhou J, Ma K, Peng Z, et al. Relationship between nocturia and hypertension: findings from the NHANES 2005-2016. *Front Cardiovasc Med*. 2023;10:1165092.doi: 10.3389/fcvm.2023.1165092

21.	Song J, Ke B, Fang X. Association of nocturia of self-report with estimated glomerular filtration rate: a cross-sectional study from the NHANES 2005-2018. *Sci Rep*. 2023;13:13924.doi: 10.1038/s41598-023-39448-0

22.	Arques S. Human serum albumin in cardiovascular diseases. *Eur J Intern Med*. 2018;52:8-12.doi: 10.1016/j.ejim.2018.04.014

23.	Cornu JN, Abrams P, Chapple CR, Dmochowski RR, Lemack GE, Michel MC, et al. A contemporary assessment of nocturia: definition, epidemiology, pathophysiology, and management--a systematic review and meta-analysis. *Eur Urol*. 2012;62:877-90.doi: 10.1016/j.eururo.2012.07.004

24.	Bockenhauer D, Bichet DG. Pathophysiology, diagnosis and management of nephrogenic diabetes insipidus. *Nat Rev Nephrol*. 2015;11:576-88.doi: 10.1038/nrneph.2015.89

25.	Li J, Guo L. Association between sleep duration and albumin in US adults: a cross-sectional study of NHANES 2015-2018. *BMC Public Health*. 2022;22:1102.doi: 10.1186/s12889-022-13524-y

26.	Zhang G, Li S, Wang S, Deng F, Sun X, Pan J. The association between serum albumin and depressive symptoms: a cross-sectional study of NHANES data during 2005-2018. *BMC Psychiatry*. 2023;23:448.doi: 10.1186/s12888-023-04935-1

27.	Sogorb MA, Vilanova E. Serum albumins and detoxication of anti-cholinesterase agents. *Chem Biol Interact*. 2010;187:325-9.doi: 10.1016/j.cbi.2010.03.001

28.	Chen S, Xia HS, Zhu F, Yin GZ, Qian ZK, Jiang CX, et al. Association between decreased serum albumin levels and depressive symptoms in patients with schizophrenia in a Chinese Han population: A pilot study. *Psychiatry Res*. 2018;270:438-42.doi: 10.1016/j.psychres.2018.10.012

29.	Vaughan CP, Fung CH, Huang AJ, Johnson TMN, Markland AD. Differences in the Association of Nocturia and Functional Outcomes of Sleep by Age and Gender: A Cross-sectional, Population-based Study. *Clin Ther*. 2016;38:2386-93 e1.doi: 10.1016/j.clinthera.2016.09.009

30.	Pesonen JS, Cartwright R, Mangera A, Santti H, Griebling TL, Pryalukhin AE, et al. Incidence and Remission of Nocturia: A Systematic Review and Meta-analysis. *Eur Urol*. 2016;70:372-81.doi: 10.1016/j.eururo.2016.02.014

31.	Rohrmann S, Crespo CJ, Weber JR, Smit E, Giovannucci E, Platz EA. Association of cigarette smoking, alcohol consumption and physical activity with lower urinary tract symptoms in older American men: findings from the third National Health And Nutrition Examination Survey. *BJU Int*. 2005;96:77-82.doi: 10.1111/j.1464-410X.2005.05571.x

32.	Shiri R, Hakama M, Hakkinen J, Auvinen A, Huhtala H, Tammela TL, et al. The effects of lifestyle factors on the incidence of nocturia. *J Urol*. 2008;180:2059-62.doi: 10.1016/j.juro.2008.07.042

33.	Prezioso D, Catuogno C, Galassi P, D'Andrea G, Castello G, Pirritano D. Life-style in patients with LUTS suggestive of BPH. *Eur Urol*. 2001;40 Suppl 1:9-12.doi: 10.1159/000049871

34.	Haidinger G, Temml C, Schatzl G, Brossner C, Roehlich M, Schmidbauer CP, et al. Risk factors for lower urinary tract symptoms in elderly men. For the Prostate Study Group of the Austrian Society of Urology. *Eur Urol*. 2000;37:413-20.doi: 10.1159/000020162

35.	Moon S, Chung HS, Yu JM, Ko KJ, Choi DK, Kwon O, et al. The Association Between Obesity and the Nocturia in the U.S. Population. *Int Neurourol J*. 2019;23:169-76.doi: 10.5213/inj.1938062.031

36.	Kim JS, Chung HS, Yu JM, Cho ST, Moon S, Yoo HJ. Analyzing the Factors Associated With Nocturia in Older People in the United States. *Ann Geriatr Med Res*. 2018;22:184-8.doi: 10.4235/agmr.18.0044

37.	Alvarez GE, Beske SD, Ballard TP, Davy KP. Sympathetic neural activation in visceral obesity. *Circulation*. 2002;106:2533-6.doi: 10.1161/01.cir.0000041244.79165.25

38.	Furukawa S, Sakai T, Niiya T, Miyaoka H, Miyake T, Yamamoto S, et al. Obesity and the prevalence of nocturia in Japanese elderly patients with type 2 diabetes mellitus: The Dogo study. *Geriatr Gerontol Int*. 2017;17:2460-5.doi: 10.1111/ggi.13103

39.	Chiu AF, Huang MH, Wang CC, Kuo HC. Higher glycosylated hemoglobin levels increase the risk of overactive bladder syndrome in patients with type 2 diabetes mellitus. *Int J Urol*. 2012;19:995-1001.doi: 10.1111/j.1442-2042.2012.03095.x

40.	Curtis LJ. An economic perspective on the causal explanations for the socioeconomic inequalities in health. *Rev Panam Salud Publica*. 2018;42:e53.doi: 10.26633/RPSP.2018.53

41.	Rubach A, Balasubramaniam K, Storsveen MM, Elnegaard S, Jarbol DE. Healthcare-seeking with bothersome lower urinary tract symptoms among men in the Danish population: the impact of lifestyle and socioeconomic status. *Scand J Prim Health Care*. 2019;37:155-64.doi: 10.1080/02813432.2019.1608412
