# Supplementary material for: Association between nocturia and serum albumin in the U.S. adults from NHANES 2005–2012
Source: PLoS One. 2024 Aug 15;19(8):e0308980. doi: 10.1371/journal.pone.0308980 (PMC11326611; doi:10.1371/journal.pone.0308980)
Supplement: Attachment — Submitted filename: Response to Reviewers.docx [file pone.0308980.s002.docx]

We would like to sincerely thank the editor and reviewers for their thorough evaluation of my manuscript. Their valuable feedback and constructive criticism have played a crucial role in refining the quality of the article. I am grateful for their dedicated efforts during the review process and for taking their comments seriously and making responsible revisions to my work.

**Editorial comment**

Question 1：Line 117–120 State that participants were screened for nocturia using a structured questionnaire. The inquiry posed was, "During the past 30 days, how many times per night did you most typically get up to urinate from the time going to bed at night to waking up in the morning? “It is not clear whether the first urine passed on waking up was included or excluded from making the diagnosis of nocturia. There is a need to clarify this.

**Our** response：We have included additional details to clarify that "the first urine passed on waking up was excluded from making the diagnosis of nocturia," which enhances the clarity of our diagnostic criteria and prevents ambiguity. (line 102-103)

Question **2:** Line21: Delete keyword “cross-section study”

**Our response:** we removed the keyword to make the article's key points more prominent. (line 20)

Question **3:** Line40, line 281: Replace the word "link" with "association."

**Our response:** We have revised the title to better align with the journal's formatting requirements. (line 24, line 264)

Question **4：**line94: change the title "Materials and Methods" to "Methods."

**Our response：**We have revised the title to better align with the journal's formatting requirements. (line 77)

Question **5:** line 145-146, line 331: Write the meaning of HDL, LDL and LUTS before the abbreviation.

**Our response:** We have corrected these errors and the explanation for the abbreviation "LUTS" has been provided in the introduction section. (line 52, line 125-126). Additionally, we made similar revisions in the abstract to better align with academic writing standards. (line 27, line 38)

**Question 6:** line 156, line 268, line 280, line 283, Reference the previous research

**Our response:** Based on previous research results, we found a significant association between serum albumin and nocturia. (DOI: 10.3390/jcm10132838, 10.1111/j.1743-6109.2012.02738.x) (line 136) Additionally, we incorporated confounding factors into our study by referencing previous research on nocturia in the NHANES database. (DOI: 10.3389/fcvm.2023.1165092, 10.3389/fpubh.2023.1186848) Furthermore, we cited relevant literature to provide a stronger foundation for our discussion. (DOI: 10.1111/j.1743-6109.2012.02738.x, 10.3390/jcm10132838) (line 264, line 267)

Question **7:** Line 284, line287: Replace the word " investigation" with "association."

**Our response:** We have revised the vocabulary to make our expression more precise and better aligned with academic standards. (line 267, line 271)

Question **8:** Line353: Replace the word "several" with "some."

**Our response:** We have revised the vocabulary as requested to ensure a more objective and clear expression. (line 333)

**Review Comments**

**Reviewer 1:**

Question 1：Figure 2 Relationship between nocturia and albumin in Model 3. Mention it paragraph.

**Our** response: We have specifically described the negative correlation between nocturia and serum albumin in Figure 2 within the relevant paragraphs, making our results more visually intuitive (lines 219-222).

Question **2:** Abbreviation in abstract without clarify. Rewrite them with journal style. References rewrite them with journal style too.

**Our response:** We have clarified the abbreviations in the abstract and updated the format of the References section to meet the requirements of PLOS ONE. (lines 27, 38, line 373-499).

**Reviewer 2**

Question 1: Line 66 reference 2: the reference shows only correlation with the QOL and not mortality.

**Our response:** We apologize for the oversight in not including relevant references in our manuscript. We have now cited the appropriate literature to support our expressions (DOI: 10.1016/j.juro.2010.09.108). (line 51)

Question **2:** Line 67 I suggest using complain or presentation rather than symptom which was repeated in the next line.

**Our response:** We have revised "symptom" to "complain" as requested, which improves the clarity and coherence of the expression. (line 52)

Question **3：**Line 86 reference 14; The patients with OAB have significantly lower SHIM score, testosterone level, and serum albumin level, have more proportion of severe ED. Lower not higher.

**Our response:** We apologize for this oversight. We have corrected the error by changing "higher" to "lower" to accurately reflect the results of previous study. (line 68)

Question **4：**Line 201 do you mean exclusion criteria?

**Our response:** We apologized for the lack of clarity in our expressions. The term "criteria" refers to the exclusion criteria. We have revised the text to avoid ambiguity (lines 183-184). The specific exclusion criteria are detailed in lines 89-95.

Question **5：**Line 370 I think good is more appropriate than excellent.

**Our response:** We have revised the vocabulary, which has made our expression more objective. (line 349)

**Reviewer 3**

Question **1:** The manuscript being presented in its current format is acceptable for publication in the journal PLOS ONE. The findings of the study and the thorough explanation of this particular topic is adequate.

**Our response:** Thanks for your positive feedback. We are pleased to hear that the manuscript is acceptable for publication in PLOS ONE and that the findings and explanation of the topic meet the journal's standards.
